# Preoperative Noninvasive Mapping Allowed Targeted Concomitant Surgical Ablation and Revealed COVID-19 Infection

**DOI:** 10.1155/2021/6651361

**Published:** 2021-03-05

**Authors:** Constantin Mork, Luca Koechlin, Matthias Streif, Alexa Hollinger, Martin Siegemund, Friedrich Eckstein, David Santer

**Affiliations:** ^1^Department of Cardiac Surgery, University Hospital Basel, Basel, Switzerland; ^2^Department of Radiology, University Hospital Basel, Basel, Switzerland; ^3^Intensive Care Unit, University Hospital Basel, Basel, Switzerland; ^4^Department of Clinical Research, University of Basel, Basel, Switzerland

## Abstract

In March 2020, a 64-year-old female with mitral valve insufficiency and persistent atrial fibrillation underwent preoperative noninvasive mapping for developing an ablation strategy. In the computed tomography (CT) scan, typical signs of COVID-19 were described. Since the consecutive polymerase chain reaction (PCR) test was negative, the severely symptomatic patient was planned for urgent surgery. Noninvasive mapping showed that atrial fibrillation was maintained by left atrial structures and pulmonary veins only. On admission day, the preoperative routine COVID-19 PCR test was positive, and after recovery, uneventful mitral valve repair with cryoablation of the left atrium and pulmonary veins was performed. Our case describes the potential benefit of preoperative noninvasive mapping for the development of a surgical ablation strategy, as well as the challenges in managing urgent surgical patients during the COVID-19 pandemic and the corresponding diagnostic relevance of CT.

## 1. Introduction

For concomitant surgical ablation, full Cox-Maze IV is the gold-standard set; however, most cardiac surgeons still prefer isolated pulmonary vein and left atrial ablation to reduce time of surgery [1]. Preoperative noninvasive mapping is a new low-risk option to plan targeted surgical ablation, which might limit surgical ablation to the maintaining sources of atrial fibrillation as well as improve efficiency and outcome of the procedure.

## 2. Case Presentation

**Figure 1:**
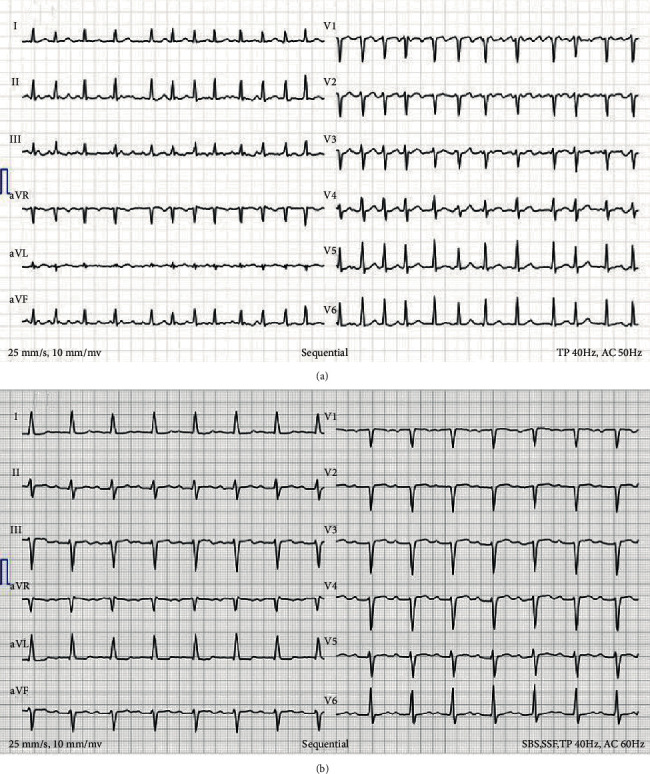
(a) The first detection (January 2020) of fibrillation with tachycardia at 142 bpm, a small QRS complex, and normal QT interval. (b) At discharge, the patient showed a sinus rhythm with an AV block I° (PQ = 264 ms), 88 bpm, small QRS complex, and normal QT interval.

We report the case of a 64-year-old woman who had been suffering from dyspnoea, dry cough, and New York Heart Association III for one and a half years and presented to the emergency department in January 2020 with dysphasia and sensory disturbances in the right hand and a left side facial paresis. A computed tomography (CT) scan revealed an acute ischaemic media infarct, wherefore lysis was administered immediately. Anticoagulation was continued with apixaban (Eliquis®, 2 × 5 mg/d, Bristol-Myers Sqibb SA, Steinhausen, Switzerland). In the electrocardiogram (ECG), a de novo persisting tachycardiac atrial fibrillation (AF; Figure 1(a)) was detected. Transoesophageal echocardiography (TTE) showed a left ventricular ejection fraction of 60% with flail leaflet of the posterior mitral valve with tendon rupture, resulting in a severe mitral valve insufficiency with an extrinsic jet and backflow into the pulmonary veins (peak gradient: 8 mmHg; mean gradient: 3 mmHg). The coronary arteries were free from any relevant stenoses. The Heart Team recommended minimally invasive surgical mitral valve repair via right anterolateral thoracotomy, concomitant surgical ablation, and closure of the left atrial appendage after completion of neurologic rehabilitation. In February 2020, the patient was transferred to the emergency department because of a new onset sensory disturbance in the right hand. Magnetic resonance imaging (MRI) disclosed a small new infarct in the medial thalamus. After neurological evaluation, the secondary prophylaxis was continued with apixaban. After discharge from neurological rehabilitation, the patient denied any neurological symptoms and was therefore referred for minimally invasive mitral valve repair and concomitant surgical ablation to the Department of Cardiac Surgery, University Hospital Basel, Switzerland, in March 2020.

**Figure 2:**
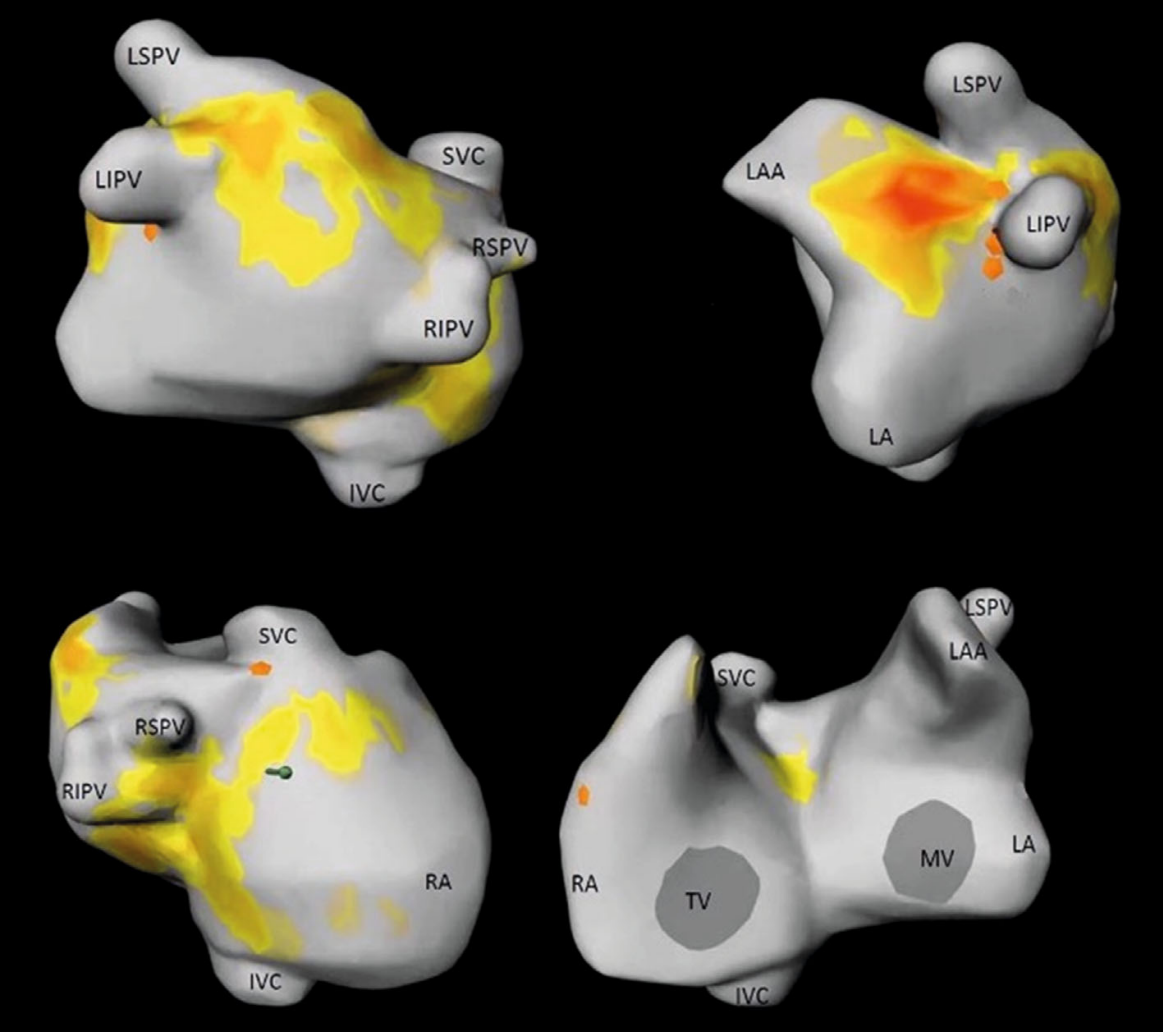
Preoperative noninvasive mapping with CardioInsight™-3D Mapping technology. Noninvasive mapping showed rotors and foci originating mainly from the pulmonary veins and left atrium; the right atrium seemed to be free from major sources of AF. IVC: inferior vena cava; LA: left atrium; LAA: left atrial appendix; LIPV: left inferior pulmonary vein; LSPV: left superior pulmonary vein; MV: mitral valve; RA: right atrium; RIPV: right inferior pulmonary vein; RSPV: right superior pulmonary vein; SVC: superior vena cava; TV: tricuspid valve.

**Figure 3:**
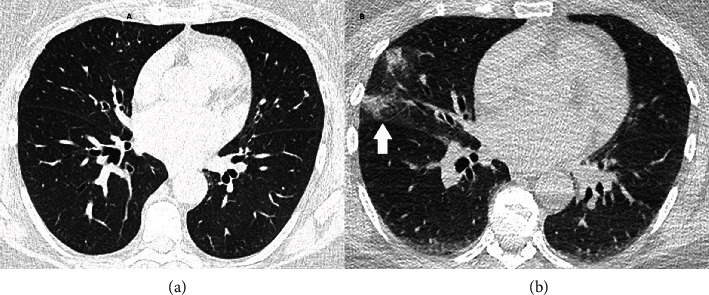
(a) CT thorax scan (December 2019) showed inconspicuous lung emphysema in comparison with the preoperative (b) CT scan (March 2020) with typical ground glass opacities (arrow) in the medial lobe.

For defining an ablation strategy, preoperative noninvasive mapping with CardioInsight™-3D Mapping technology (CIT, Medtronic SA, Münchenbuchsee, Switzerland) was performed in our outpatient clinic. This noninvasive mapping system collects chest ECG signals and combines these signals with CT scan data to produce and display simultaneous, biatrial and biventricular three-dimensional (3D) cardiac maps. A 3D epicardial image of the patient's atria was created with localization of foci and rotors of AF. Noninvasive mapping showed rotors and foci originating mainly from the pulmonary veins and the left atrium. The right atrium was clear (Figure 2). Furthermore, in this preoperative CT scan, pure ground glass opacities with a consolidation typical for COVID-19 were observed in the left lung (Figure 3(a) and (b)) [2]. The patient reported persisting dry cough, dyspnoea, and intermittent elevated temperature over the past few weeks. However, these were the same symptoms she had been experiencing for the past months and therefore could be explained by symptomatic mitral valve insufficiency. Nevertheless, the patient was requested to undergo immediate polymerase chain reaction (PCR) test (SARS-CoV-2 Reverse Transcription Quantitative Nucleic Acid Testing (RT-QNAT) targeting specific viral sequences of the spike-glycoprotein S-gene (Basel SCo V2-S-111 bp)) [3], which was performed by the general practitioner the day after the CT scan. Since the PCR result was negative, the patient was admitted for surgery seven days later. On March 26, 2020, the day of admission, the patient was normothermic, and the oxygen saturation was 96%. However, routine preoperative SARS-CoV2 PCR test was positive, and the patient was transferred to the COVID-19 isolation ward. Because she was asymptomatic, she was discharged from the hospital one day after to undergo home quarantine. On April 8, 2020, after two negative SARS-CoV2 test results, uneventful minimally invasive mitral valve repair with concomitant endocardial pulmonary vein isolation, left atrial cryoablation (CryoICE cryoprobe, Atricure Europe B.V., Amsterdam, The Netherlands), and endocardial suture of the left atrial appendage was performed via right anterolateral minithoracotomy. After surgery, the patient was transferred to the intensive care unit and underwent continuous atrial pacing for 24 hours. She was extubated on the day of the operation, transferred to the cardiac surgical ward on the second postoperative day, and discharged into rehabilitation on postoperative day 7. At discharge, the TTE showed a normal ejection fraction (60%), with a minimal residual mitral valve insufficiency. The patient was in sinus rhythm with first degree AV block (Figure 1(b)). After three months (July 2020), TTE showed a normotrophic left ventricle with an ejection fraction of 60% and mild mitral valve regurgitation. Holter ECG-detected sinus rhythm with a heart rate of 81 beats per minute was observed, with a small QRS complex and no detection of atrial fibrillation. The patient denied dyspnoea and palpitations, and the persisting dry cough had also stopped. In the lung function test, pulmonary static and dynamic volumes and capacities were normal. The residual restricted haemoglobin-corrected diffusion capacity was interpreted in the context of a postviral condition. In comparison to January 2020, there had been an improvement of the forced expiratory volume (+680 ml), the total lung capacity (+470 ml), and the diffusion capacity (+12%) in July 2020. The X-ray also showed a regression of the ventilation disorder. Neurologically, the patient had recovered very well with slightly discrete pyramidal stroke residuals in the right arm. She denied cognitive impairment and problems with alertness or language deficits.

## 3. Discussion

This case describes the option of targeted surgical ablation by preoperative noninvasive mapping, as well as the complexity in managing a patient with symptomatic mitral valve regurgitation and signs of a COVID-19 infection in a preoperative CT scan with a false-negative COVID-19 test. We want to underline four main messages from this case: (1) The decision of when to operate on urgent cardiac surgical patients with a recent or active COVID-19 infection is difficult to reach during an evolving pandemic. Recommendations for cardiac surgery during COVID-19 have been published suggesting not deferring surgery in case of severe mitral valve regurgitation [4]. Yet, since the patient was stable, we decided to defer the operation for another two weeks after the positive COVID-19 test result so that she could recover from her infection. However, the operation could have certainly not been postponed. If the patient would have been unstable, the surgery would have been performed as recommended [4]. (2) The Basel SCoV2- S-111 bp test with an analytical sensitivity and specificity of 99.68% (95% CI, 95%–100%) and 98.99% (95% CI, 91%–100%), respectively, was performed [3]. However, the CT scan offered a more reliable result than the standard PCR test [5]. Since the patient has been suffering from dry cough for over 18 months due to her mitral valve insufficiency, and since the primary PCR test was false-negative for unknown reasons, the COVID-19 infection was perfectly masked. (3) Although the full Cox-Maze IV is the gold-standard set for surgical ablation, most surgeons prefer isolated pulmonary vein and left atrial ablation to reduce complexity and cardiopulmonary bypass time [1]. Therefore, in our specific case, preoperative noninvasive mapping offered efficient preoperative guidance, which allowed performing targeted ablation on existing rotors and foci. According to our noninvasive mapping results, a full Cox-Maze IV with right atrial ablation retrospectively could be considered overtreatment. After three months, the patient is still in sinus rhythm with complete cardiopulmonary and neurological recovery. (4) Finally, in our case, the preoperative CT revealed unexpected but essential data and probably reduced the risk for postoperative complications. This shows again that preoperative routine CT scan should be discussed prior cardiac surgery [6]. Before the patient underwent a CT scan, the reasonable explanation for her symptoms was the progressive course of the mitral valve insufficiency. Therefore, the patient was prioritized for surgery in the first place despite the COVID-19 pandemic. Retrospectively, it can be seen that the COVID-19 infection might have been responsible for the accentuation of the symptoms and was masked by the symptomatic mitral valve disease.

In winter 2020, the uncontrolled COVID-19 pandemic is still ongoing, and common viral infections are upcoming during the winter months. Therefore, although testing tools have tremendously improved within the last few months, the challenge to rule out concomitant COVID-19 infection in periods of seasonal concomitant viral infections such as the flu and in the context of comorbidities with similar symptoms remains high. In conclusion, the current pandemic demands an increased awareness for cardiac symptoms which might be either masked or exacerbated by COVID-19 and vice versa.

## Data Availability

The datasets used and/or analysed during the current study are available from the corresponding author on reasonable request.
